# Spontaneous cancer remission after COVID-19: insights from the pandemic and their relevance for cancer treatment

**DOI:** 10.1186/s12967-023-04110-w

**Published:** 2023-04-21

**Authors:** Concetta Meo, Giuseppe Palma, Francesca Bruzzese, Alfredo Budillon, Claudio Napoli, Filomena de Nigris

**Affiliations:** 1 Department of Precision Medicine, School of Medicine, University of Campania “Luigi Vanvitelli”, Via De Crecchio 7, 80138 Naples, Italy; 2 S.S.D. Sperimentazione Animale, Istituto Nazionale Tumori - IRCCS - Fondazione G. Pascale, Naples, Italy; 3 Scientific Directorate - National Institute of Cancer - IRCCS - Fondazione G. Pascale, Naples, Italy; 4 Clinical Department of Internal Medicine and Specialistic Units, Division of Clinical Immunology and Immunohematology, Transfusion Medicine, and Transplant Immunology (SIMT), Azienda Universitaria Policlinico (AOU), 80138 Naples, Italy; 5 Advanced Medical and Surgical Science (DAMSS), School of Medicine, University of Campania “Luigi Vanvitelli”, 80138 Naples, Italy

## Abstract

Early in the COVID-19 pandemic, it emerged that the risk of severe outcomes was greater in patients with co-morbidities, including cancer. The huge effort undertaken to fight the pandemic, affects the management of cancer care, influencing their outcome. Despite the high fatality rate of COVID-19 disease in cancer patients, rare cases of temporary or prolonged clinical remission from cancers after SARS-CoV-2 infection have been reported. We have reviewed sixteen case reports of COVID-19 disease with spontaneous cancer reduction of progression. Fourteen cases of remission following viral infections and two after anti-SARS-CoV-2 vaccination. The immune response to COVID-19, may be implicated in both tumor regression, and progression. Specifically, we discuss potential mechanisms which include oncolytic and priming hypotheses, that may have contributed to the cancer regression in these cases and could be useful for future options in cancer treatment.

## Introduction

The management of COVID-19 mortality in cancer patients is gradually improving, but many questions regarding the impact of COVID-19 on cancer remain. The Eastern Cooperative Oncology Group (ECOG) established that morbidity and mortality are greater in subjects with an ascertained COVID-19 diagnosis and cancer than in cancer free patients [1, 2]. In particular, lung cancer and hematological malignancies have poorer outcome following infection than other cancers [1–6]. This is probably due to a reduced respiratory capacity and more severe immune suppression in patients with these malignancies. Data linking oncologic treatment and cancer prognosis after COVID-19 remain difficult to evaluate. Complicating factors include the various treatment regimens for both cancer and COVID-19, the temporal relationship between cancer stages and COVID-19 exposure, and patient-specific confounders. However, despite the lethality of malignant tumors and increased risk following COVID-19 some spontaneous cancer remission have been reported in the absence of cancer-specific treatment. Clinical cases of remissions in tumors following viral infections are not new [7]. One of the earliest report of the beneficial effect of a viral infection described the remission of leukemia by influenza [7, 8]. Some lymphoma cases were reported subsequently [7–10]. Whether COVID-19 disease may counteract the natural cancer history has not been systematically investigated. A better understanding of the immunogenic impact of COVID-19 and its effect on cancer mechanisms would help to optimize therapeutic strategies in such patients. Here, we evaluated fourteen cases of COVID infection and two cases of vaccination associated with spontaneous cancer retardation. Although a more prolonged follow-up will be needed, we discuss cancer care, including examination, diagnosis, surgery, and provide a framework of potential mechanisms that may have contributed to the cancer regression and exposed patients to upcoming cancer complication.

### SARS-CoV-2 and hematological malignancies

#### Classical hodgkin lymphoma—case 1

A case of a patient positive for Epstein-Barr virus (EBV) affected by classical Hodgkin lymphoma at stage IIIS confirmed by PET/CT was reported by Challenor and colleagues [11]. This patient resulted positive for SARS-CoV-2r before starting any treatment, as shown in Table 1. The patient received supportive ward-based care for COVID-19, without corticosteroid or immune-chemotherapy, and was discharged after 11 days to convalesce at home. During follow-up without tumor treatment, 4 months later, the patient showed reduced lymphadenopathy monitored by PET/CT scan. Furthermore, metabolic uptake was reduced and EBV viral load decreased from 4800 to 413 copies/mL, indicative of remission. On the base of hematological analysis, the authors hypothesized that the mechanisms of lymphoma regression could be due to cross-reactivity of pathogen-specific T cells with tumor antigens determining cell death and massive activation of inflammatory cytokines following infection [11].

**Table 1:** Baseline characteristics of tumor, stratified by patient, treatment and response post-COVID-19 infection/vaccine

Case n°	Pathology	Patient age (years)/Sex	Treatment	Covid-19/ Vaccine	Follow-up	References
1	Classical hodgkin lymphoma	61/Male	No corticosteroid or immune-chemo-therapy was administered.	Covid-19	Four months after COVID-19 diagnosis: complete remission	[11]
2	Acute myeloid leukemia	57/Female	NO chemotherapy for AML. COVID-19 was treated with remdesivir at 200 mg intravenously (IV) on the first day, followed by 100 mg daily for 5 days and dexamethasone IV at 8 mg daily for 10 days. Trasfusion with six units of packed red blood cells and ten units of platelets. Pirfenidone, colchicine (0.6 mg twice daily), and oxygen therapy	Covid-19	Six months after, diagnosis of COVID-19complete remission after 8 months later relapse	[12]
3	Acute myeloid leukemia	63/Female	Postpone treatment protocol. Supportive therapy only (Fluconazole 150 mg 3 times daily/Azithromycin 500 mg daily and Prednisone 100 mg for 5 days)	Covid-19	After 5 weeks from COVID-19 diagnosis remission and one year later no evidence of disease	[13]
4	T-ALL	28/Male	COVID-19 supportive treatment (Azithromycin 500 mg/day and Prednisone 40 mg daily for 5 days)	Covid-19	After 6 weeks diagnosis of COVID-19: no evidence of disease	[13]
5	Advanced follicular lymphoma	79/Female	Without lymphoma treatment	Covid-19	Nine months later diagnosis of COVID-19: complete remission	[14]
6	Follicular lymphoma	61/Male	NA	Covid-19	After 5 months from diagnosis of COVID-19: complete remission	[15]
7	Relapsed/refractory NK/T-cell lymphoma associated with Epstein-Barr virus (EBV) and autoimmune hemolytic anemia (AIHA)	20/Male	Red-blood cell transfusions and methylprednisolone at rate of 1 mg/kg of body weight per day were administered. No antiviral or cloroquine drugs were administered	Covid-19	Transient remission of NK lymphoma: remission during COVID-19 infection, followed after 1 month : lymphoma relapse.	[16]
8	Multiple myeloma	76/Female	A single dose of filgrastim, levofloxacin and acyclovir were administered. After the decreasing blood-oxygen levels, the patient was treated with dexamethasone and remdesivir.	Covid-19	During the next 2 months from COVID-19 diagnosis: no evidence of multiple myeloma	[17]
9	Chronic lymphocytic leukaemia (CLL)	67/Male	Asymptomatic untreated CLL. Multiple treatments including intravenous immunoglobulin and broad-spectrum antibiotics were used for febrile neutropenia	Covid-19	Complete remission after 12 months of follow-up after diagnosis of COVID-19	[18]
10	Metastatic renal cell carcinoma cancer (mCRC)	71/Male	Aside from supportive measures, no specific treatment for COVID-19 was administered.	Covid-19	Follow-up on 6 months from COVID-19 diagnosis: partial regression of metastatic lesions	[19]
11	Metastatic renal cell carcinoma cancer (mCRC)	58/Male	Antibiotics; firstly, clarithromycin and, subsequently, amoxycillin clavulanate for persistent fever. No antineoplastic treatment.	Covid-19	Four months later COVID-19 diagnosis: reduction in lung metastases	[19]
12	Metastatic colorectal cancer (mCRC)	65/Male	FU/FA/BEVA /depotentiated treatment, with the last cycle performed 1 month before COVID-19 infection	Covid-19	Follow-up at 18 months: from COVID-19 diagnosis complete response with regression of hepatic lesions	[20/21]
13	Metastatic colorectal cancer (mCRC)	58/Male	The patient refused post-operative chemotherapy.	Covid-19	Post COVID-19 infection: reduction of the liver metastatic lesion. Time to-Progression: 23 months	[20/21]
14	Metastatic colorectal cancer (mCRC)	60/Female	After six cycles with FOLFIRI/PANI, CT scan in September 2020 displayed disease stabilization	Covid-19	Progression of disease at 2 months from diagnosis of COVID-19	[21]
15	Primary cutaneous anaplastic large-cell lymphoma	57/Male	NA	COVID-19 vaccination (BionTech/Pfizer)	1 week before the second dose of COVID-19 vaccination: complete resolution of untreated lymphoma	[22]
16	Myoepithelial carcinoma of the left parotid and lung lesion	61/Female	Close surveillance.	Covid19 vaccination (mRNA-1273)	Persistent tumor shrinkage on follow-up of 9 months, after the second dose of the vaccine.	[23]

#### Acute leukemia—case 2/case 3/case 4

A 57-year-old woman with the initial diagnosis of AML M2, showed that 65% of the bone marrow space was occupied by 60% of blasts. Additionally a stronger reduction of megakaryocytes and erythroid precursors were observed. During the first few days of hospitalization, she resulted positive for COVID-19 and received treatment with Remdesivir at 200 mg intravenously (IV) and dexamethasone for 10 days, as showed in Table 1. Fifteen days later a CT scan evidenced a notable progression compared to admission in bilateral alveolar opacities. After 2 months after the initial diagnosis, the blood cell counts improved and bone marrow aspirate analysis revealed a normocellular marrow composed of 55% cells among them less than 5% were blasts and trilineage maturation. Flow cytometry analysis indicated the presence of 3% myeloblast cells in the absence of disease-specific therapy for acute leukemia. However, 8 months after the first diagnosis, the marrow test revealed the presence of recurrence of the primary disease [12].

A 63-year-old female patient diagnosed COVID-19 infection by polymerase chain reaction (PCR) showed a blood picture with 30% blast cells, 7.3 g/dL hemoglobin, and platelet count of 82 × 10^3^/µL without lymphopenia, as shown in Table 1. Bone Marrow Aspiration (BMA) analysis revealed the presence of 53% blast cells, dysplasia in the erythroid and myeloid series, and was diagnosed as acute myeloid leukemia. However, the leukemia treatment protocol was postponed until the recovery from the viral infection, and the patient received supportive therapy based on (Fluconazole 150 mg 3 times daily, Azithromycin 500 mg daily, and Prednisone 100 mg for 5 days). Five weeks after discharge PCR nasopharyngeal swabs were negative, and the cancer diagnostics confirmed the myelodysplastic syndrome and established refractory cytopenia with trilineage dysplasia. The exam of BMA showed that blast cells dropped from 53% to only 3% [13].

In another case, a 28-year-old male affected by T acute lymphoblastic leukemia (T-ALL) for 6 years developed COVID-19 infection. The nasopharyngeal swab positive test and the CT multiple bilateral ground-glass appearances confirmed the infection diagnosis, as shown in Table 1. During the infection, the patient presented multiple cervical lymphadenopathies, blood cytometry examination indicated anemia, absolute lymphocytosis, and 30% blast cells. However, lymphocyte immunophenotyping revealed an atypical increased expression of major histocompatibility complex class II (MHC class II). The patient was treated with azithromycin and prednisone (40 mg/daily) for 5 days. Two weeks later, PCR was negative for Sars-Cov-2 virus. Six weeks after the start of COVID-19 infection, the cervical lymphadenopathy disappeared and blood flow cytometry was negative for atypical cells. No relapse occurred during follow-up (at 5 and 12 months). The authors speculated that stimulation of the immune response against COVID-19 infection could have caused anti-tumor immunity in these patients, or the virus may have exerted an oncolytic activity on tumor cells [13].

#### Follicular lymphoma case 5/case 6

A 79-year-old woman with skin follicular lymphoma after resection of lesion stayed in remission for 3 years, then she developed localized relapse. PET/CT revealed follicular lymphoma with breast and bone manifestations without structural lesions. As the patient was asymptomatic and had a low disease burden, she was subjected to a watch and wait approach. At the same time, COVID-19 was diagnosed with positive serology. Nine months later and without further lymphoma treatment, PET/CT revealed the resolution of widespread lesions, which led the authors of the case report to assume a connection between SARS-CoV-2 infection and remission [14].

In another case, at the end of treatment with (18)FDG-PET/CT, a 61-year-old male with follicular lymphoma showed bilateral pneumonia due to SARS-CoV-2 infection confirmed by PCR on nasal swab, as shown in Table 1. A comparison of images taken at the time of COVID diagnosis with baseline revealed a reduced para-aortic lymph node lesion, suggesting a partial response to chemotherapy agent R-bendamustine. After SARS-CoV-2 recovery, two consecutive biopsies were negative and FDG-PET-CT scan did not reveal metabolic activity, suggesting complete follicular lymphoma remission. Sollini et al. speculated that the SARS-CoV-2 infection triggered an immune response, first inducing what they call a local “*flare phenomenon*” deductible from an increase in the size of the nodal lesion, followed by the “*abscopal effect*” [15].

#### Relapsed/Refractory NK/T-cell lymphoma—case 7

Pasin and colleagues reported a transient remission of NK/T cell lymphoma during COVID-19 disease, as showed in Table 1. A 20-year-old male presented with a history of relapsed/refractory NK/T-cell lymphoma associated with Epstein-Barr virus infection and autoimmune hemolytic anemia (AIHA). The NK/T-cell lymphoma did not respond to treatment with multiple, immuno-chemotherapy protocols. Then, the patient developed COVID-19 diagnosed by the presence of diffuse bilateral ground-glass at CT and positivity to nasopharyngeal swab test. During the first 10 days, the administration of red blood cells, steroids, oxygen, intravenous levofloxacin, and supportive therapy achieved only a partial increase in hemoglobin level, platelet count, and hemolytic markers. Subsequently, an unexpected spontaneous steady clinical improvement was documented. There was an increase in hemoglobin levels, hemolytic markers, and platelet counts. A reduction in leukocyte count, NK cells, and plasma EBV-DNA copies were decreased. Spleen enlargement was reduced. The clinical and laboratory results suggested a remission of NK lymphoma during COVID-19. Unfortunately, the NK/T-cell lymphoma relapsed on day 34 after a negative oropharyngeal COVID-19 swab. The patient showed a rapid return of hemolytic anemia, fever, spleen enlargement, and an increase in NK cell count, and the plasma was positive for the Epstein-Barr virus’s DNA. The authors considered this case to be a transient remission of NK/T cell lymphoma after SARS-CoV-2 infection [16].

#### Multiple myeloma—case 8

A 76-year-old woman with a diagnosis of multiple myeloma (MM) received a CyBorD treatment (cyclophosphamide, bortezomib, and dexamethasone) due to impairment of her renal function. After a single cycle of MM therapy, serum proteins analysis revealed a significant reduction of the free k light chain, and the patient developed a SARS-CoV-2 infection, confirmed by a positive PCR test. She was then started on a 10 day course of Dexamethasone (6 mg) and Remdesivir (200 mg once followed by 100 mg daily for 4 days) plus a single unit of convalescent plasma, as shown in Table 1. After an improvement in her respiratory status, the patient was discharged. Seven weeks later, laboratory data showed substantial improvement in myeloma disease markers and renal function. Moreover, 4 months after the end of MM therapy and COVID-19, the patient was still in cancer remission. The authors hypothesize that the pharmacologic agents used during the treatment of COVID-19 disease interrupted signaling pathways necessary for myeloma cell proliferation. Additionally, the administration of convalescent plasma has conferred a humoral immunity that may have contributed to preventing MM progression or augmented the cytotoxic effects of CyBorD, resulting in complete remission [17].

#### Chronic lymphocytic leukemia—case 9

A 67-year-old male with untreated asymptomatic chronic lymphocytic leukemia (CLL) for 8 years exhibited proliferation in peripheral blood of monotypic B-lymphocytes, which constituted 89% of lymphocytes. The patient showed multiple lymphadenopathies in bilateral neck and axillary regions. A CT scan also revealed multiple enlarged lymphadenopathies in the chest. In addition, ground-glass opacities associated with bilateral and multi-lobar consolidation foci confirmed the COVID-19 positivity. The patient had not previously received a COVID-19 vaccine, as shown in Table 1. One week after admission, the patient developed pancytopenia and a high fever. Two weeks later, he was negative for SARS-CoV-2 virus but cytopenia and prolonged fevers > 38 °C persisted for 2 months. Bone marrow aspirate analysis indicated a normal distribution of erythroid and myeloid cells. However, the bone marrow cellularity was 80%, due to diffuse infiltration of both CD4^+^ and CD8^+^ T lymphocytes. Five months after COVID-19, whole-body PET/CT detected no characteristic hypermetabolic focus, regression of mediastinal lymphadenopathies, and the patient’s blood did not show proliferation of monotypic B-lymphocytes. Longer-term follow-up indicated no typical clinical presentations of CLL (lymphocytosis, lymphadenopathy, hepatomegaly, splenomegaly), and the patient was still in complete remission 12 months after recovery from COVID-19. The authors believe that the infection could have triggered immune responses against tumor cells [18].

### SARS-CoV-2 and solid tumors

#### Renal cell carcinoma—case 10/case 11

Buchler et al. reported two cases of spontaneous regression of metastases from renal cell carcinoma (mRCC) after COVID-19, as shown in Table 1. In the first case, a CT scan of a 71-year-old male revealed a kidney tumor with marked mediastinal lymphadenopathy and multiple lung metastases. A few weeks after diagnosis patient developed COVID-19, but no specific treatment was administered. After the end of the quarantine period, a nephrectomy was carried out. A CT scan performed 3 months after COVID-19 diagnosis surprisingly showed marked regression of all metastatic tumors. Consequently, no further cancer treatment was initiated, and a follow-up CT scan a further 3 months later confirmed continued substantial regression of metastatic lesions [19].

The second patient was a 58-year-old male with PCR-confirmed the presence of SARS-CoV-2 virus. The patient was started on antibiotics. A post-COVID-19 CT scan showed lung parenchymal changes but also detected a left renal mass and multiple lung lesions indicating metastatic cancer. Two months later, a nephrectomy was carried out but the patient received no systemic anti-neoplastic treatment. Four months after the COVID-19 diagnosis PET/CT images showed a reduction in lung metastases [19].

#### Colorectal cancer—case 12/case 13/case 14

Three patients affected by metastatic colorectal cancer (mCRC) had a reduction of disease burden during COVID-19, as shown in Table 1. In a 65-year-old man with colon adenocarcinoma and liver metastases CT scan revealed disease stabilization after 7 months of first-line chemotherapy (FOLFOX/BEVA), followed by three cycles of maintenance treatment (FU/FA/BEVA). However, the month following the last cycle, the patient developed severe COVID-19 were as CT images registered complete regression of hepatic lesions.

The second patient was a 58-year-old man with colon adenocarcinoma and three unresectable liver metastases. He was treated for 6 months with pre-operative FOLFOX/BEVA, followed by multiple metastasectomies and post-operative chemotherapy. 23 months later, a CT scan documented the progression of hepatic lesions. Concomitantly the patient developed mild symptomatic COVID-19. After a month, a new CT image indicated a disappearance of the liver metastasis [20].

Patient 3 was a 60-year-old woman who received a left hemicolectomy for adenocarcinoma, followed by 6 months of adjuvant FOLFOX therapy, as shown in Table 1. PET/CT scans illustrated an increase in disease and multiple measurable nodules on the peritoneal surface as well as a small nodule in the right lung. After six cycles of first-line chemotherapy (FOLFIRI/PANI), she achieved disease stabilization and concomitantly presented symptoms associated with COVID-19. A CT scan 2 months after COVID-19 remission showed an unexpected reduction of peritoneal and lung disease. The authors hypothesized a correlation between SARS-CoV-2 infection and tumor burden reduction [21]. Moreover, assessment of genetic landscapes evidenced common mutation in BARD1 gene (p.Val507Met) in cases 12 and 13 [20, 21]. BARD1 mutation is a germline alteration associated with higher risk of some heritable form of cancers [21]. Because in affected lymphocytes (lymphomas) this mutation is associated with greater resistance to apoptosis this may account for greater lymphocyte survival to viral infection, and consequently activation against cancer, as in this case of CRC shrinkage.

### SARS-CoV-2 vaccination and cancer

#### Cutaneous anaplastic large-cell lymphoma—case 1

Gambichler and colleagues reported the case of a 57-year-old male patient with a recurrent primary cutaneous anaplastic large-cell lymphoma (pcALCL) and diffuse lung manifestation, as showed in Table 1. Initially, pcALCL showed frequent local relapses predominantly in the scalp and neck. Treatment with methotrexate, brentuximab, gemcitabine, and radiotherapy) led to a remission of cutaneous lesions, while a pathologically enlarged cervical lymph node and innumerable bilateral pulmonary nodules were noted. Histopathology of lung lesions revealed infiltrates of Hodgkin/Reed–Sternberg-like cells. Before initiation of brentuximab therapy and 1 week after having received the first COVID-19 vaccination (Comirnaty, BioNTech/Pfizer, Mainz, Germany), the ultrasound and thoracic CT of the patient showed shrinking of the cervical lymph node and resolution of the diffuse lung lesions. Due to the tight timeline, the authors speculated that the SARS-CoV-2 vaccine was the causal factor of the marked spontaneous regression from pcALCL [22].

#### Myoepithelial carcinoma of the parotid case 2

A 61-year-old woman affected by myoepithelial carcinoma of the left parotid was diagnosed by CT scans with bilateral metastatic lung involvement after para-thyroidectomy and postoperative radiotherapy to the left neck, as shown in Table 1. Given the absence of curative treatment for metastases of this cancer and the lack of standard systemic therapy for myoepithelial carcinoma, it was decided to proceed with close surveillance. Ten months after the initial diagnosis, on January 2021 the patient received the first dose of mRNA-1273 COVID-19 vaccine (*Moderna*) and a CT scan at the same time showed a significant increase in the size of the pulmonary nodules. However, after the second vaccine dose in February 2021, a CT scan of the chest showed 13% shrinkage of the pulmonary nodules. Subsequently, follow-up CT scans demonstrated persistent tumor shrinkage: 50%, 67%, and 73% reduction at 3, 6, and 9 months, respectively after the second dose of the vaccine. Therefore, the authors hypothesized that the tumor shrinkage was attributable to a systemic inflammatory response induced by the COVID-19 vaccine [23].

### Cancer remission hypothesis

#### Priming hypothesis

**Fig. 1:**
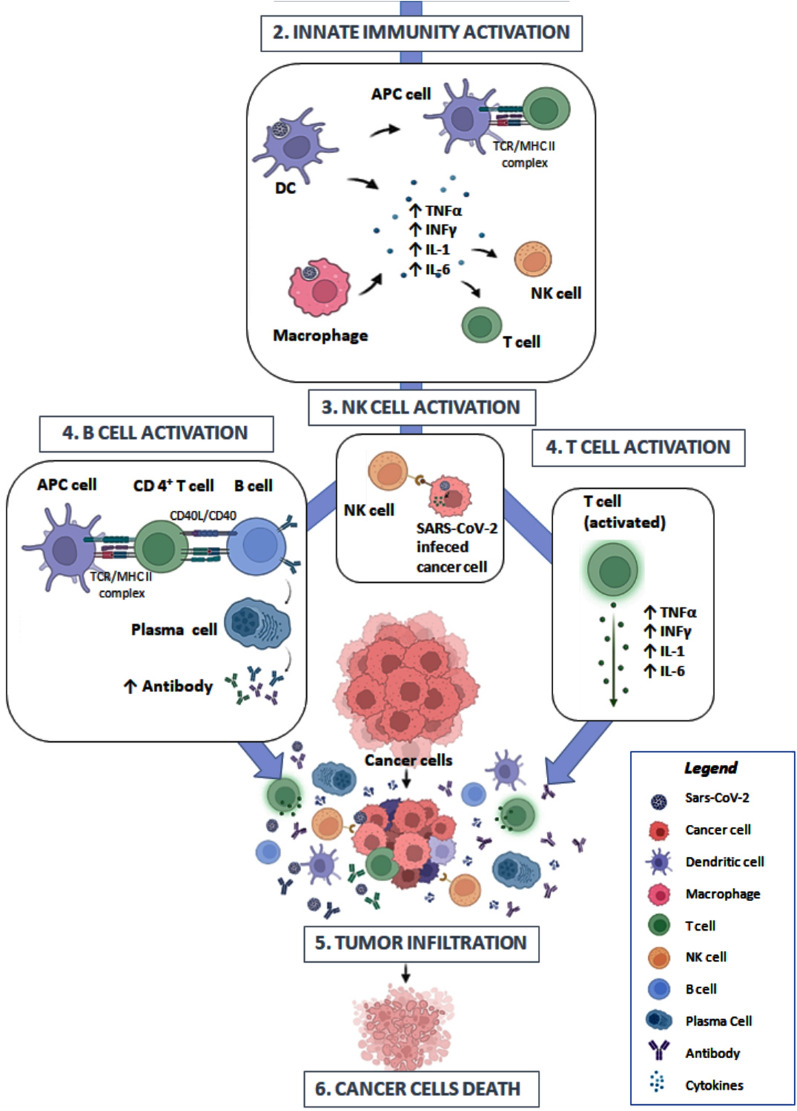
Priming hypothesis: (1) The infection by SARS-CoV-2 induced a severe acute inflammatory response through innate immunity activation. (2) The immune cells including macrophages and dendritic (DC), in response to SARS-CoV-2 start the production of inflammatory cytokines, including interleukins IL-6, IL-1, TNF-α and INF-γ, that hyperactivate (3) NK and T cells. (4) Furthermore, Antigen-Presenting Cells (APC cells) efficiently prime CD8 + cytotoxic T cells, CD4 + T helper cells and B cell maturation, (5) maintaining an activated state of tumur-infiltrating lymphocytes and (6) promoting the death of cancer cells *The figure was created with BioRender.com*

The microenvironment of solid cancers is rich in CD4^+^FOXP3^+^ regulatory T cells (Treg), which suppresses immune responses, and thus contribute to the development and progression of tumors [24]. The metabolism of cancers may also may lead to T-cell exhaustion, a form of T-cell dysfunction characterized by progressive loss of critical death effector function [25]. It emerged that infection induced a severe acute inflammatory response (innate immunity activation). In addition, common clinical symptoms of COVID-19, such as fever and extended sequelae of respiratory symptoms, suggest that efficient T-cell priming triggered by prolonged inflammation may contribute to increased activation of adaptive immune responses against malignant growth (Fig. 1). Evidence from other infections occurring in tumour patients attributes anti-cancer effects to inflammation and strong infiltration by lymphocytes macrophages and dendritic cells (DCs) [26, 27]. The heightened inflammatory response associated with SARS-CoV-2 infection is well known [28]. The underling mechanism would be consequence of SARS-CoV-2 replication in immune cells (innate immunity) infected via ACE2 receptors. The viral RNA is recognized by immune intracellular receptor, such as toll-like receptors (TLR3, TLR7, and TLR8) [29]. Cells then start the transcription via NFKβ of inflammatory cytokines, including interleukins IL-6, IL-1, TNF-α and INF-γ, that hyperactivate T cells [30]. These cytokines play an important role by promoting and maintaining an activated state of tumour-infiltrating lymphocytes [31]. Blood-based tests show the characteristic signs of inflammation, the systemic antiviral response driven by T-cell, macrophage rich inflammation and IFNγ production [32]. Yet, SARS-CoV-2 specific CD4^+^ T cells directed against the S protein have been detected in survivors of severe COVID-19 disease suggesting a direct virus priming [33]. Indeed, in some cases of spontaneous SARS-CoV-2 eradication and acceleration of tissue repair an efficient priming of CD8^+^ cytotoxic T cells, CD4^+^ T helper cells and B cell maturation through the appropriate orchestration of Toll-like receptor 2 and interferon signalling have been reported [34]. In the cases of solid tumor temporary remission following vaccination the histological examination clearly revealed an increase of tumor infiltration by CD8^+^, CD4^+^ T cells, granzyme B^+^ cytolytic cells, and antigen-presenting cells [21–23]. This is supported by the findings that immune-dependent mechanisms secondary to COVID-19 associated to overwhelming production of proinflammatory cytokines can potentially destroy tumor cells by overcoming an immunosuppressive tumor microenvironment in solid tumors [19–23]. Another important question is whether Sar-Cov-2 virus promotes the recovery of immune function of cytotoxic T-cells seen in patients treated with protein 4 (anti-CTLA-4) or anti-PD-1/PD-L1 [35]. During COVID-19 disease, the immune checkpoint blockage might be naturally induced, since SARS-CoV-2 spike proteins are predicted by computational analysis to interact with, and block PD-1 signalling, similar to pembrolizumab and nivolumab-like monoclonal antibodies favouring the activity of the CD8 T killer cell population [36]. However, these pathways may also contribute to tumour progression. Proinflammatory cytokines (IL-1, IL-6, IL-8, and TNF-α), activated by COVID-19 are known to drive tumorigenesis via NFkΒ and KRAS pathways [37, 38]. The IL-6/JAK/ STAT signaling pathway is central to tumorigenesis and formation of an immunosuppressive tumor microenvironment [39]. Moreover, the chronic inflammation and oxidative stress can lead to DNA damage promoting carcinogenesis [40, 41] and immune checkpoint signalling [42]. Thus, immune dysregulation influences COVID-19 severity and may have either positive or negative effects during anti-tumor immune responses. Future large studies investigating the balance of molecular pathways determining regression or progression of cancer will have to verify which one prevails.

#### Oncolytic hypothesis

**Fig. 2:**
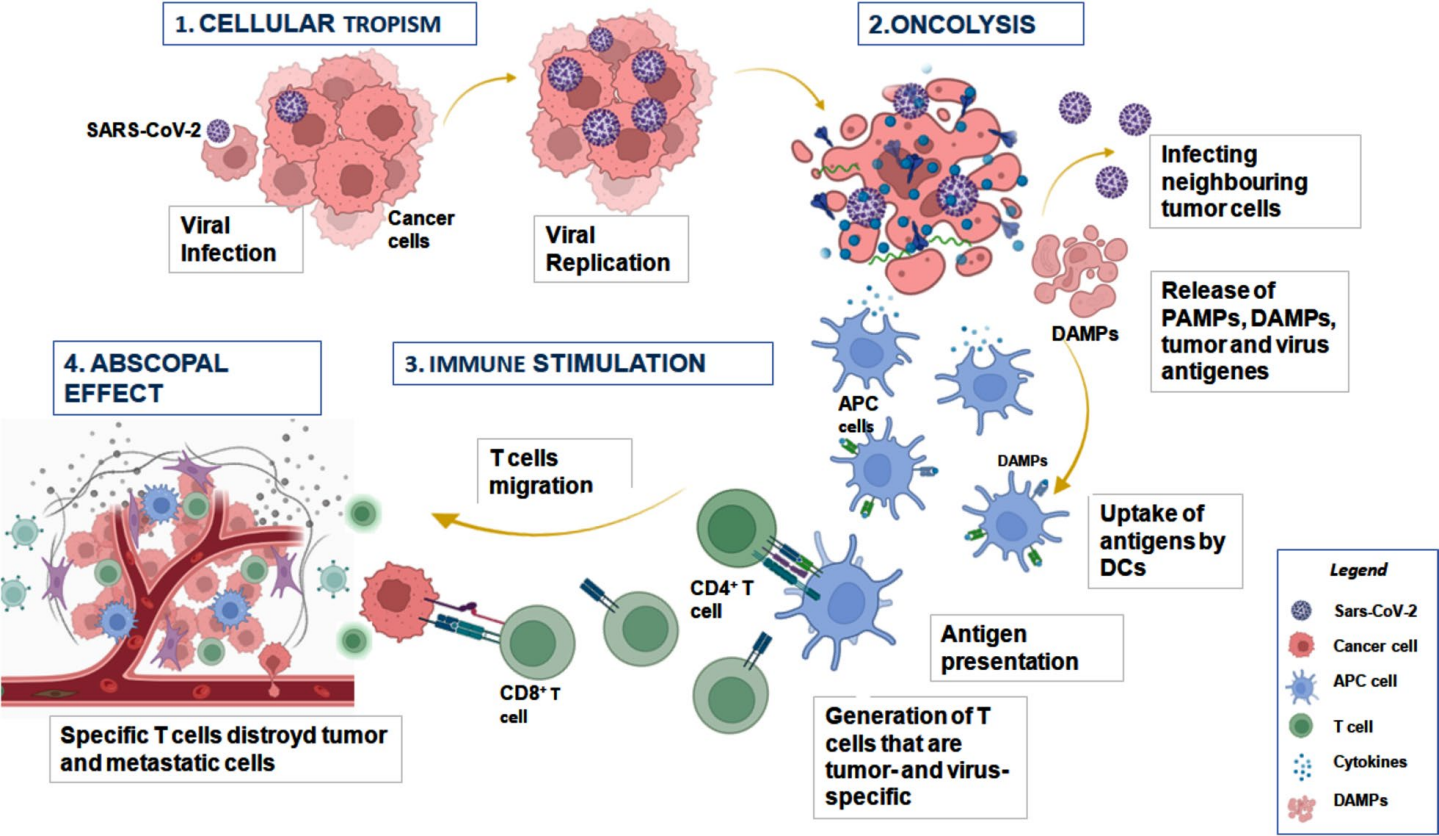
Oncolytic viruses effects: (1) The OV is an organism able to identify and infect cancer cells (cellular tropism), replicate massively in the cancer cells until to promote the lytic phase with release of the tumor-associated antigen (TAAs). (2) The release pathogen-associated molecular patterns (PAMPs) and damage-associated molecular patterns (DAMPs) from the tumor cells. (3) Dendritic cells then migrate to lymph nodes and present the antigens to the T cells. The TAAs stimulate the immune system of the host against cancer cells and (4) the T-cells can also effect elimination of metastasized tumors, in the abscopal effect. *The figure was created with BioRender.com*

An alternative hypothesis to explain cancer remission relies on the assumption that SARS-CoV-2 may act as an oncolytic virus, as suggested by the NK lymphoma case reviewed here [16] (Fig. 2**)**. The general mechanism established for oncolytic viruses is their ability to target specific cells, to use their hosts’ proliferative machinery to replicate themselves and then to destroy their host cells. Cellular lysis occurs when the cell’s resources are completely depleted and it can no longer produce viral particles. The cell selectivity of virus infection is determined by different factors as the absence of a clear architecture of the tissue, high permeability membrane and the increased expression of molecules that serve as receptors for the penetration of the virus into the cell [43, 44]. It is well known that SARS-CoV-2 mainly targets the lung, heart [45], brain [46], kidney [47], gut [48], but also NK cells that express the ACE2 receptor [21]. It is reasonable to assume that the virus can also infect tumor cells in these organs. SARS-CoV-2 penetrates host cells by two main routes: direct penetration of its genome into the cytosol via fusion with the host cell membrane, or endocytosis [49], followed by replication and transcription of the viral genome [50]. The infected cells may then undergo pyroptosis, a process of inflammatory cell death and subsequent release of damage-associated molecular patterns (DAMPs), such as viral nucleic acids and oligomers [51]. This mechanism may further stimulate the immune response of the host against infected cancer cells, thus causing partial tumor regression [52]. Evidence exists that virus protein ORF3a blocks the initial microtubule nucleation, inhibiting cell cycle and causing apoptosis of infected cells [36]. This result is also supported by an increase of apoptosis in patients following infection [53, 54]. SARS-CoV-2 easily infects NK cells that massively express ACE2. Following lysis NK cells decline in numbers and lose immune function [16, 52]. Indeed the viral copy number of EBV, a sensitive biomarker of NK/T cell lymphoma, was markedly reduced during the course of COVID‑19 in one of the cancer regression cases [16]. Moreover additional observations indicated that open reading frame 8 (ORF8), from SARS-CoV-2 down-regulating MHC class I, reduced the activation of NK and CD8 cells [55, 56]. Other targets of Spike protein have been predicted by bioinformatic and computational analyses. Studies predicted that SARS-CoV-2 spike protein might enter into lymphoma cells via specific surface markers (CD15, CD27, CD45, CD152) [36]. The interactions between SARS-CoV-2 proteins and the ICAM-1 receptor on MM cells induce lysis of MM and CD138^+^ plasma cells [17]. More recently, computational modelling also demonstrated that CD147 antigen present on several tumor cells is a potential binding protein of COVID-19 infection [57]. Blocking of this receptor may activate ferroptosis in tumor cells and indicate CD147 as a potential target of virotherapy [58]. However, further pathogenic investigations of the interactions between SARS-CoV-2 and cancer cells are necessary to firmly establish the mechanisms responsible for the oncolyticeffect. At the same time, a potentially oncogenic role of SARS-CoV-2 has been proposed; possibly due to dysregulation of the cell cycle leading to the cellular transformation. The Nsp3 and Nsp15 of SARS-CoV2 may contribute to the degradation of tumour suppressor proteins, e.g. P53 or retinoblastoma (pRb) [59, 60]. Finally, the S2 subunit of SARS‐CoV‐2 may also interact with P53 and BRCA1/2 [61]. In conclusion, SARS-CoV-2 infection shows a double face. By its potential ability to promote either regression or progression of cancer, it may improve or worsen the prognosis. The cumulative effect of coronavirus infection on cancer remains to be elucidated.

Genetic engineering of viruses with oncolytic activity is rapidly growing as a novel therapeutic approaches to advanced form of cancer refractory to first line of treatment. Currently several trials of such viruses in combination with immune checkpoint inhibitors are in progress. Talimogene laherparevec (T-VEC) is the first engineered oncolytic herpes simplex virus (HSV) that received United States Food and Drug Administration approval for the treatment of advanced melanoma [62, 63]. T-VEC is a double-edged weapon that not only destroys the target tumor cells at the site of injection but could also initiate a localized and systemic immune response promoting death of tumor cells [64]. Dendritic cells then present viral antigens to T-helper cells and cytotoxic T-cells and thereby mount a huge immune response at the systemic level [64]. Moreover T-VEC, in combination with immune check point inhibitors such as ipilimumab (an inhibitor of the immune-suppressor protein, CTLA-4), has entered clinical trials for melanoma (https://clinicaltrials.gov/ct2/show/NCT01740297). Similarly, the efficacy of T-VEC in combination with pembrolizumab (a monoclonal antibody that blocks another immune checkpoint protein, PD-1) to improve the outcome of melanoma is currently under investigation (https://clinicaltrials.gov/ct2/show/NCT02263508).

## Conclusion

It is well known that cancer patients have one of the highest risks of severe COVID-19 complications and mortalities [1, 2]. Viral infections can greatly affect the host immune system of cancer patients by mechanisms beyond our current knowledge. An obvious difference in the adaptive immune system was observed in the majority of cancers [65–67] and many comorbidities and immune-senescence have worsened the outcome [68–70]. The cases of tumor regression reported here represent a small number, compared to the large numbers who have died during the pandemic, although some novel cases are emerging [71]. These cases comprise a great variety of parameters, such as different tumors and stages, different cancer types and COVID-19 treatments, different co-morbidities, and no long follow-up data are currently available. Nevertheless, the results showed that despite immunosuppression some patients with onco-hematological diseases and cancer developed at least temporary remission after infection. The mechanism responsible could involve unexpected immune responses, oncolytic activity of virus, or both. The activation of immune-responses in onco-hematological patients is confirmed by recent successful results from SARS-CoV-2 vaccination. In fact, in a large cohort of patients activation of T-cells and serological immunity indicate that residual immunity function can be activated by DAMPS [72]. Data reported in the present paper are clearly too varied to draw scientific conclusions with any statistical power. However, they did provide thought-provoking details and working hypotheses for such future large studies. For example COVID-19 treatments, such as Dexamethasone and Remdesivir may interrupt signaling pathways needed to maintain tumor proliferation. Dexamethasone, in particular, inhibits secretion of inflammatory cytokines by bone marrow stromal cells, such as IL-6 required for the growth of multiple myeloma (MM) cells [30], suggesting that immunotherapy against IL-6 could be helpful as adjuvant to treat MM. Additionally, the observation that, immune-compromised cancer patients, develop acute inflammatory response following infection indicates that the immune system can be activated by strong inflammatory events [19–21]. Chemokine storm and cell mediate immunity worsen COVID-19 severity, whereas in a few cases of cancer regression showed an efficient priming of cell-mediated immune response and oncolytic activity. For example, in two cases immunocompromised patients experienced intense reactogenic response after two doses of COVID-19 mRNA vaccine [23, 22] and showed oncolytic activity in NK lymphoma [16]. Another face of the coin are the molecular pathways activated by COVID-19 (NFkΒ, JACK-STAT and INF-1γ signaling that are often altered by both solid and hematopoietic malignancies and may therefore promote tumor cell proliferation instead of resumption of normal immune responses [73]. These aspects should be considered when attempting to reduce post-infection side effects in cancer patients. More patients, prolonged follow up and further investigations will clearly be needed to understand the molecular pathways and possible clinical relevance of interactions between COVID-19 and cancer. The cases cited provide support for the assumption that infections in the respiratory tract initiated immune responses against systemic cancer even in tumors refractory to other treatment. Oncolytic viruses should be therefore considered for the possible treatment of immunologically repressed cold tumors resistant to other immunotherapy, e.g. immune checkpoint blockades and chimeric antigen receptor T cells. Challenges remain to select a virus capable of replicating in cancer cells, generating an immune-activating microenvironment and leading to antigen spreading [58]. The rapid progress in the development and worldwide use of mRNA vaccines increases the chances of using mRNA against SARS-CoV-2 for cancer patients. Although methods to optimize the structure of mRNA vaccines, their stability and delivery methods are advanced, additional clinical development is needed for cancers, but if successful, could improve future treatment options for malignancies.

## Data Availability

Not applicable.
